# HPV16 L1 and L2 DNA methylation predicts high-grade cervical intraepithelial neoplasia in women with mildly abnormal cervical cytology

**DOI:** 10.1002/ijc.28050

**Published:** 2013-01-21

**Authors:** Attila T Lorincz, Adam R Brentnall, Nataša Vasiljević, Dorota Scibior-Bentkowska, Alejandra Castanon, Alison Fiander, Ned Powell, Amanda Tristram, Jack Cuzick, Peter Sasieni

**Affiliations:** 1 Queen Mary University of London, Centre for Cancer Prevention, Wolfson Institute of Preventive Medicine, Barts and The London School of MedicineLondon, EC1M 6BQ, United Kingdom; 2 Obstetrics and Gynaecology, School of Medicine, Cardiff UniversityHeath Park, Cardiff, CF14 4XN, United Kingdom

**Keywords:** HPV, DNA methylation, cervical cancer, triage, biomarkers

## Abstract

DNA methylation changes in human papillomavirus type 16 (HPV16) DNA are common and might be important for identifying women at increased risk of cervical cancer. Using recently published data from Costa Rica we developed a classification score to differentiate women with cervical intraepithelial neoplasia grade 2 or 3 (CIN2/3) from those with no evident high-grade lesions. Here, we aim to investigate the performance of the score using data from the UK. Exfoliated cervical cells at baseline and 6-months follow-up were analyzed in 84 women selected from a randomized clinical trial of women undergoing surveillance for low-grade cytology. Selection of women for the methylation study was based on detectable HPV16 in the baseline sample. Purified DNA was bisulfite converted, amplified and pyrosequenced at selected CpG sites in the viral genome (URR, E6, L1 and L2), with blinding of laboratory personnel to the clinical data. The primary measure was a predefined score combining the mean methylation in L1 and any methylation in L2. At the second follow-up visit, 73/84 (87%) women were HPV16 positive and of these 25 had a histopathological diagnosis of CIN2/3. The score was significantly associated with CIN2/3 (area under curve = 0.74, *p* = 0.002). For a cutoff with 92% sensitivity, colposcopy could have been avoided in 40% (95% CI 27–54%) of HPV16 positive women without CIN2/3; positive predictive value was 44% (32–58%) and negative predictive value was 90% (71–97%). We conclude that quantitative DNA methylation assays could help to improve triage among HPV16 positive women.

**What's new?**

The human papillomavirus (HPV) genome is subject to changes in DNA methylation. Here, the quantification of DNA methylation in HPV16 from exfoliated cervical cells of women with detectable virus was found to be significantly associated with cervical intraepithelial neoplasia grade 2 or 3. The methlyation score was based on the combined mean of methylation specifically in the HPV16 late regions (L1 and L2). The approach could be used to aid decisions concerning triage to colposcopy and has the potential to be expanded to other carcinogenic HPVs.

Dynamic methylation of DNA and proteins is the main molecular mechanism underpinning epigenetics. It plays an important role in cancer and may be the predominant way that genotype interacts with the environment.^1^,^2^ Human genes and the human papillomavirus (HPV) genome are subject to large changes in DNA methylation during carcinogenesis, and these levels can be accurately measured using relatively simple and inexpensive assays that show promise for diagnosis and prognosis.^3^–^6^

Cervical cancer is caused by persistent infection with one of approximately a dozen high-risk (HR) HPV types. Testing for HR-HPV DNA is likely to become the predominant method for cervical screening in the near future due to its high sensitivity.^7^ However, the main drawback of HR-HPV testing is a relatively lower specificity than for cytology. To compensate for this limitation different triage algorithms have been suggested, including use of cytology and p16 immunochemistry^8^,^9^ which both require the use of a specimen that preserves morphology. An alternative is a molecular triage test that would allow HR-HPV-positive women to be reflex-tested from the original screening specimen; this is particularly attractive in some situations such as in vaginal self-collection approaches.^10^ Genotyping for HPV16 and HPV18 has been proposed as a triage test of HR-HPV positives to improve specificity, however, better specificity comes at the cost of a relatively large drop in sensitivity of 50% or more for cervical intraepithelial neoplasia grade 2/3 or cancer (CIN2+). A related problem is that the positive predictive value (PPV) of HPV16 genotyping is usually less than 25%; therefore, a substantial majority of women referred to colposcopy on the basis of an HPV16-positive result will not have CIN2+. Meanwhile the HR-HPV-positive women not referred to colposcopy still need careful follow-up within 1 year.^11^ The low PPV for HPV genotyping has prompted an ongoing search for better molecular triage tests. The goal is to provide an accurate determination of disease state and to improve the cost-effectiveness of HPV-based screening by avoiding colposcopy and frequent follow-up for most HR-HPV infected women.^9^–^12^

Quantitative measurement of DNA methylation shows promise as a simple test for the diagnosis and prognosis of many cancers. In women infected by HPV16, the levels of methylation increase slowly with duration of HPV persistence and increase more dramatically with the diagnosis of cervical cancer.^13^,^14^ These observations open up the possibility of accurately predicting which women will develop cancer years in advance. Of more immediate interest to clinicians looking for efficient management strategies, a study of women from Costa Rica looking at methylation levels in the viral late regions, specifically CpG 6457 in L1 demonstrated the detection of prevalent CIN2+ among HPV16 infected women with a sensitivity of 90% and a specificity of 60%.^13^ The efficiency of L1 methylation triage appeared to increase with age; in women above the median age in the study (28 years) at a sensitivity of 90% the specificity increased to more than 75%.^13^

Using pilot data from a Costa Rican screening population,^13^ we developed three classifier scores based on methylation of selected CpG sites in HPV16 L1, L2, URR and E6 open reading frames (ORFs). Classifiers 1 and 2 were developed to identify women with CIN grade 2/3 while classifier 3 was developed to predict persistence of HPV infection. In the current study, we measured the methylation of relevant CpG sites and applied the three classifiers with an aim to validate the predefined scores in the UK based cohort of women under surveillance due to presence of low-grade cervical abnormalities.^15^

## Material and Methods

### Study population—UK clinical trial

**Figure 1:**
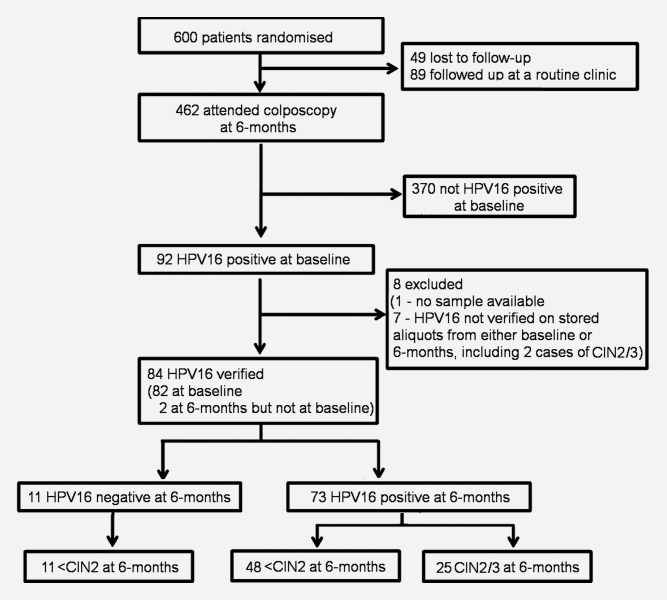
Consort Diagram describing the selection of patients in the UK cohort.

The study was conducted according to REMARK guidelines for assessing biomarker test performance.^16^ Relevant details of the cohort have been described previously.^15^ In brief, the study was designed primarily as a double-blind, randomized controlled trial of 150 mg “diindolylmethane” (DIM) or placebo daily for 6 months in women with newly diagnosed, low-grade (borderline changes or mild dysplasia) cytological abnormalities; this classification group is broadly equivalent to a combination of low-grade squamous intraepithelial lesion or atypical squamous cells of undetermined significance in the Bethesda System.^17^ Randomization was in the ratio 2 (DIM) to 1 (placebo). All women were invited for colposcopy at 6 months with biopsy of any abnormality. The study protocol was reviewed and approved by the South East Wales Local Research Ethics Committee (Ref# 03/5093) and informed written consent was obtained from each participant before randomization. An independent Data and Safety Monitoring Committee was in place throughout the trial to review study progress. The trial is registered at http://ClinicalTrials.gov (number NCT00462813) and ISRCTN (number 47437431). Samples from this clinical trial are well suited to our aims of molecular investigation of DNA methylation because there was standardized collection of exfoliated cervical cell specimens in SurePath™ (Becton Dickinson, Sparks, MD) vials at both baseline and at the 6-month follow-up just before colposcopy. The study did not reveal any significant effects of DIM on either HPV infection or cervical morphology and so we do not consider this aspect further.^15^ The CONSORT diagram is presented in Figure 1.

### Test endpoints

The primary endpoint of this study was presence of histologically confirmed CIN2/3 6–12 months after enrolment. No cancers were found in our study. The secondary endpoint was HPV16 status on a sample taken at the 6-month visit. Cytology and histological samples were read and reported within the routine Cervical Screening Wales program. Women for the study were selected because they were HR-HPV positive on their baseline sample by PCR-enzyme immunoassay (PCR-EIA) using GP5+/6+ primers performed at the HPV laboratory, Cardiff University School of Medicine.^18^ Presence of HPV16 DNA was confirmed in a second PCR-EIA, using individual oligo-nucleotide probes. Methylation results are only reported if the aliquot tested in the Molecular Epidemiology Laboratory at the Centre for Cancer Prevention, Queen Mary University of London, confirmed the presence of HPV16 DNA either on the methylation assay or by quantitative real time PCR in E6 ORF.^19^

### DNA isolation and bisulfite conversion

Genomic DNA was extracted from cervical specimens with QIAamp DNA Mini Kit (Qiagen, Hilden, Germany) and measured as previously described.^20^ DNA (250 ng) was used in the bisulfite conversion reactions where unmethylated cytosines were converted to uracil with the EZ DNA methylation kit (Zymo research, Irvine, CA) according to manufacturer's instructions.

### HPV16 DNA methylation assay

Sites indicating diagnostic and/or potential to predict persistence in L1, L2, E6 and URR in the Costa Rican cohort^13^ were selected for development of the classifier scores. Therefore, methylation was measured in the sites constituting the scores, namely CpG-L1 6367, 6389; CpG-L2 4238, 4247, 4259, 4268, 4275; CpG-E6 218, 220, 245; and CpG-URR 31, 37, 43, 52, 58 in baseline and 6-month follow-up specimens from the UK clinical trial. Briefly, PCRs were performed using 1.2–1.5 µl of converted DNA equivalent of 1,500 cells using the PyroMark PCR kit (Qiagen) as previously described.^13^ Thermal cycling was initiated at 95°C for 15 min, then 50 cycles: 30 sec at 94°C; 30 sec at the annealing temperature^13^; 30 sec at 72 °C, and a final extension for 10 min at 72 °C. In each run, a nontemplate negative control was run in addition to a standard curve consisting of 1 pg/µl 0, 50 and 100% methylated HPV16 plasmid in a background of 10 ng/µl human DNA. PCR product (10 µl) was pyrosequenced using a PyroMark™ Q96 ID (Qiagen) instrument. Raw pyrogram data were analyzed by the PSQ96MA software and the peak height proportions of cytosine (*C*, indicating methylation) and thymidine (*T*, not methylated) for individual CpG sites were converted into percentage methylated *C* by the formula *C*/(*C* + *T*) × 100 at each interrogated CpG site.

### Statistical methods

We set two aims in the predefined statistical analysis plan: (*i*) to validate the performance of two classifiers (S1 and S2) to separate HPV16 infected women with CIN2/3 from infected women without high-grade lesions (*ii*) to validate the ability of a classifier (S3) to predict persistent HPV16 infection. The three classifiers were developed using pilot data from the Costa Rican study (Supporting Information Report) before the analysis of the methylation data collected from UK clinical trial.

The primary classifier score (S1) for risk of CIN 2/3 was defined:


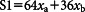


where variable (*x*_a_) denotes the proportion of methylated L2 CpG sites (a site is methylated if measurement is >0%) and can take values 0, 0.2, 0.4, 0.6, 0.8, 1.0. Variable (*x*_b_) is the average methylation of CpG 6367 and 6389 expressed as a decimal between 0 and 1. A failed methylation assay was treated as zero methylation in both variables. In Costa Rican data, at optimal cutoff S1 = 5, sensitivity was 93% and specificity 52%.

To investigate if methylation of E6 and URR would contribute valuable information in identifying presence of high-grade lesions, a secondary classifier score (S2) for risk of CIN 2/3 was defined as:


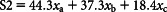


with the additional variable (*x*_c_) based on any detectable methylation in E6 and URR. (*x*_c_) is therefore 1 if any of the 8 CpG sites in E6 or URR were methylated (measurement > 0%) and 0 otherwise. In Costa Rican data, at optimal cutoff S2 = 18, sensitivity was 90% and specificity 64%. Because the S1 and S2 were developed using the methylation data collected on the last cytological sample before diagnosis in the Costa Rican study, we applied the classifiers scores 1 and 2 only on the methylation data from 6-month follow-up samples.

For development of classifier S1 and S2, the cancers in the Costa Rica data were excluded to avoid a risk of bias from the very high levels of methylation typical of these lesions and because we desired classifiers that can efficiently detect CIN2 and CIN3. However, we believe that our classifiers may also be relevant for cancers because they mostly have more extreme methylation values than CIN2/3.^13^

A third classifier (S3) was defined to investigate if methylation could be useful to predict women who were likely to be HPV16 DNA positive again after the initial positive result, *i.e*. persistence of the infection. As only methylation in L1 appeared to be predictive of persistence in the Costa Rica data, the third classifier (S3) was defined as the mean methylation of the two CpGs in L1 (*i.e*. S3 = *x*_b_). At 4% methylation cutoff in the Costa Rica data, persistence was predicted.

The performance of the three classifiers was evaluated by receiver operating characteristics (ROCs), area under the ROC curve (AUC) and logistic regression likelihood ratio statistics (*χ*^2^).^21^ A nonparametric BCa bootstrap confidence interval (5,000 replicates)^22^ was used for AUC and Wilson confidence intervals were used for sensitivity, specificity and positive/negative predictive value.^23^ The Cuzick trend test was used to investigate changes in methylation with increasing lesion severity.

In addition, the diagnostic value of cytology at the 6-month time point was assessed. Boxplots and summary statistics were used to compare methylation across CpGs. A Wilcoxon signed-rank test was used to look for significant differences in methylation between HPV16+ women at baseline and 6 months, where failed methylation was set to zero. Analyses were performed using STATA 12.0 and R 2.11.1 software packages.

## Results

### Predefined primary analysis: validation of the classifiers

**Figure 2:**
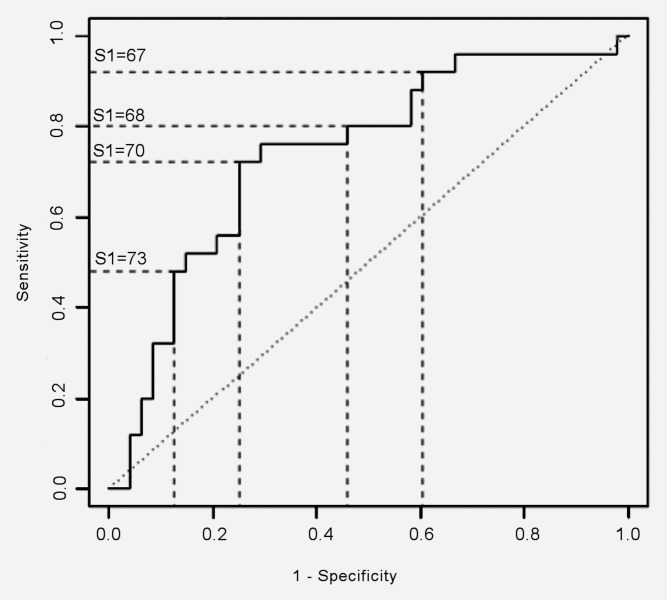
ROC curve resulting from application of classifier S1 to the 6-month samples of women with HPV16, to separate those with and without CIN 2/3. AUC = 0.74 (95% CI 0.60–0.85). Selected sensitivity and specificity points with corresponding S1 scores are shown. The sensitivity and 1-specificity values range from 0 to 1 and these correspond to percentages of 0% to 100% respectively.

Presence of HPV16 DNA was confirmed by quantitative real time PCR in 82 of 84 base line samples (the negative samples were excluded) and 73 of the 6-month samples. Of the latter, 25 women had a histopathological diagnosis of CIN2/3, where 10 were diagnosed with CIN2 and 15 CIN3. No cancers were detected in this cohort. The S1 classifier was applied to the methylation data obtained from the 73 women who were still HPV16 positive at 6 months. Overall, the S1 score showed an increasing trend (*p* = 0.0004), between <CIN2 (interquartile range (IQR) 17, 68, median 70), CIN2 (IQR 69, 70, median 73) and CIN3 (IQR 71, 74, median 77). At the predefined cut-point from Costa Rica data (S1 = 5), the S1 classifier had 96% sensitivity for CIN2/3, but a low specificity of 15% (Table 1). Figure 2 shows the ROC curve for S1 with AUC = 0.74 (95% CI 0.60–0.85) and *χ*^2^ of 9.78 (*p* = 0.002). Supporting Information Table 1 shows various points along the ROC plot with different tradeoffs of sensitivity and specificity. To account for the difference between the two investigated populations, we investigated at which cut-point a minimum of 90% sensitivity would be retained, but optimal specificity found (Table 1). Consequently, we obtained a cut-point of S1 = 67 in the UK clinical trial, which yielded sensitivity of 92% and specificity of 40% (27–54%), PPV 44% (32–58%) and negative predictive value (NPV) 90% (71–97%). At S1 = 67, two CIN3 were missed and at S1 = 5 one CIN3 was missed.

**Table 1:** The sensitivity and specificity of classifiers S1, S2 and S3 at the predefined cut-points and at cut-points with maximized specificity and sensitivity of at least 90%

Classifier (cut-point)	Number of classified CIN2/3 (total = 25)	Sensitivity (%)	Number of classified <CIN2 (total = 48)	Specificity (%)
S1 (5)	24	96	41	15
S1 (67)	23	92	29	40
S2 (18)	24	96	35	27
S2 (56)	23	92	29	40
	Number of classified persistent (total =71)		Number of classified clearer (total = 11)	
S3 (4)^1^	67	94	8	27

1 The S3 classifier did not show significant ability to predict persisitence of infection; therefore, cut-point for maximized specificity was not explored.

Next, classifier S2 was applied to the 73 HPV16 positive women at 6 months. S2 performed slightly better than S1 at the predefined cut-point (S2 = 18) but equally at the cut-point maximizing the specificity with 90% sensitivity (S2 = 56) (Table 1).

**Figure 3:**
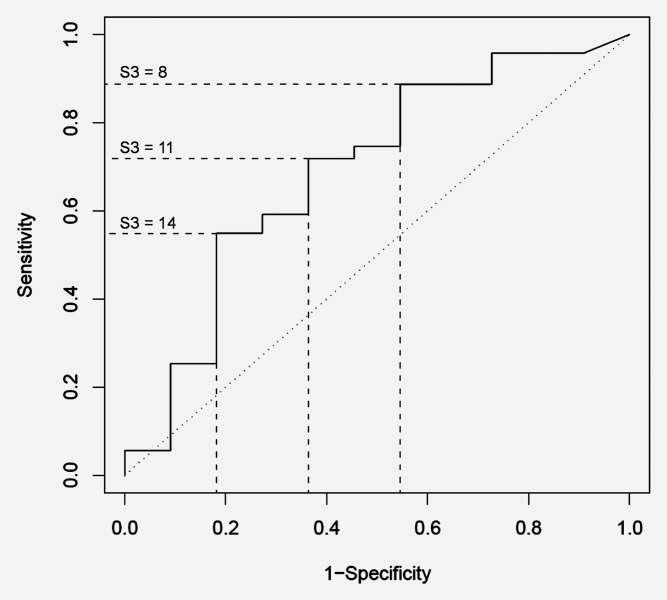
ROC curve resulting from application of classifier S3 to separate women positive for HPV16 DNA only at baseline from women positive at both time points. AUC = 0.69, *χ*^2^ = 2.7 (*p* = 0.10). Selected sensitivity and specificity points with corresponding S3 scores are shown. The sensitivity and 1-specificity values range from 0 to 1 and these correspond to percentages of 0% to 100% respectively.

Lastly, classifier S3 was applied to the 82 baseline samples, where it displayed a sensitivity of 92% and specificity of 27% at the predefined cutoff of 4 for predicting 6-month HPV16 persistence. The corresponding PPV was 89% and NPV 39%. Figure 3 shows the ROC for S3 with AUC = 0.69, and *χ*^2^ = 2.7 (*p* = 0.10). Although statistical significance was not attained this might be due to lack of power because only 11 women (12%) cleared HPV16 DNA in 6 months. Supporting Information Table 2 shows the values of sensitivity and specificity for other selected points along the ROC.

**Table 2:** Mean methylation, number and percentage of unmethylated specimens and failed assays in each region at baseline and six months

		URR				E6		
			
		Number of cases	Mean methylation^1^	Unmethylated N (%)^2^	Failed N (%)	Mean methylation	Unmethylated N (%)	Failed N (%)
First	Clear	11	1.6	3 (27)	0 (0)	2.0	3 (27)	1 (9)
sample	Persist	71	1.6	14 (20)	0 (0)	2.5	7 (10)	0 (0)
	Total	82	1.6	17 (21)	0 (0)	2.5	10 (12)	1 (1)
First	<CIN2	57	1.7	12 (21)	0 (0)	2.4	6 (11)	1 (2)
sample	CIN2/3	25	1.6	5 (20)	0 (0)	2.6	4 (16)	0 (0)
	Total	82	1.6	17 (21)	0 (0)	2.5	10 (12)	1 (1)
Second	<CIN2	48	2.3	5 (10)	1 (2)	3.2	6 (13)	2 (4)
sample	CIN2/3	25	3.0	2 (8)	0 (0)	4.2	2 (8)	0 (0)
	Total	73	2.5	7 (10)	1 (1)	3.6	8 (11)	2 (3)

		**L2**				**L1**		
			
First	Clear	11	11.0	3 (27)	2 (18)	14.2	0 (0)	1 (9)
sample	Persist	71	15.3	14 (20)	2 (3)	20.0	2 (3)	1 (1)
	Total	82	14.8	17 (21)	4 (5)	19.3	2 (2)	2 (2)
First	<CIN2	57	13.0	15 (26)	3 (5)	16.2	1 (2)	2 (4)
sample	CIN2/3	25	18.7	2 (8)	1 (4)	25.9	1 (4)	0 (0)
	Total	82	14.8	17 (21)	4 (5)	19.3	2 (2)	2 (2)
Second	<CIN2	48	17.1	10 (21)	3 (6)	20.0	0 (0)	1 (2)
sample	CIN2/3	25	22.4	1 (4)	0 (0)	25.7	0 (0)	0 (0)
	Total	73	19.0	11 (15)	3 (4)	22.0	0 (0)	1 (1)

1 Mean methylation is stated in percent

2 n (%) means number of cases and (percentage) of cases

### Cytology

Cytology results were available for 67 HPV16 positive women at 6 months, of whom 24 were CIN2/3 (Supporting Information Table 3). Cytology showed 67% sensitivity (95% CI 45–84%) and 42% specificity (95% CI 27–58%) with corresponding PPV 39% and NPV 69% if any cervical abnormality present (borderline or worse) was used as a cut-point to predict CIN2/3.

### Exploratory analysis of methylation in UK clinical trial

**Figure 4:**
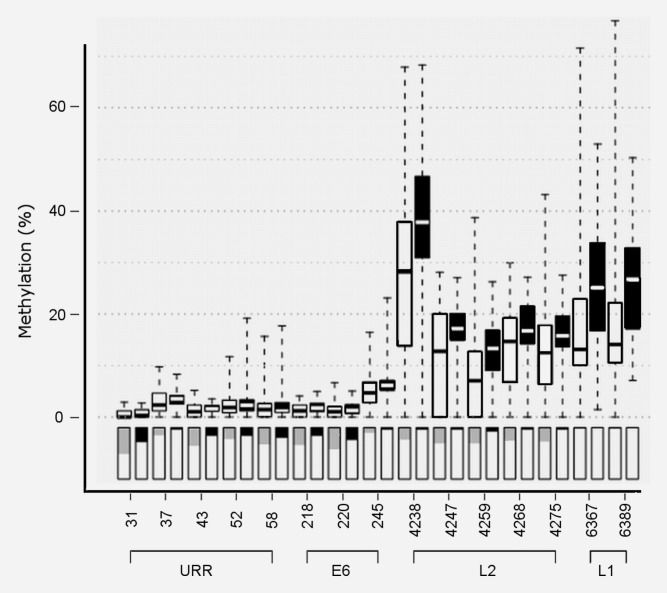
DNA methylation distribution in each investigated CpG site. The box plots show the distribution of average, lower, and upper quartile methylation values with whiskers extending to the minima and maxima. The white box plots represent methylation in 59 <CIN2 women and the black box plots represent the results for 25 women who were CIN2/3. In the corresponding boxes below the box plots, the proportion of unmethylated assays is illustrated in gray for <CIN2 and black for CIN2/3. Failed assays were excluded.

The distribution of methylation of each investigated CpG site in women with and without CIN2/3 is shown in Figure 4. This demonstrated that methylation was relatively high in the L2 and L1 regions with median methylation between 11% in CpG 4259 and 33% in CpG 4238, while methylation was very low to non-detectable in the URR and the E6 regions, *i.e*. <5%. Further, the methylation of L1 was higher in women with CIN2/3, who also had a greater proportion of sites in L2 that were methylated (Fig. 4) concurring with the S1 classifier score showing that these combined patterns had significant power to predict CIN2/3.

Mean methylation, number of unmethylated, and number failed methylation assays in each ORF at baseline and 6 months, separated by whether they cleared HPV16 infection or were diagnosed with CIN2/3 are presented in Table 2. The methylation was greater in the 6-month samples than at baseline; significantly so for L2 (average +4.2% from 71 who persisted at baseline to 73 HPV16+ at 6 months, *p* = 0.004), URR (+0.9%, *p* < 0.001), and E6 (+1.1%, *p* = 0.002), but non-significantly for L1 (+2.0%, *p* = 0.141). For persistent infections, paired differences in methylation between baseline and 6-month samples are shown in the Supporting Information Figure 1 where changes in the distributions can be more readily appreciated.

## Discussion

We have confirmed the diagnostic value of HPV16 DNA methylation classifier S1 for identifying women with CIN2/3 in a clinical study of women with low-grade cytology and persistent HPV16 infection over at least 6 months. We found that for a cutoff with 92% sensitivity, colposcopy could have been avoided in 40% (95% CI 27–54%) of HPV16 positive women without CIN2/3; PPV was 44% (32–58%) and NPV was 90% (71–97%). As a relatively low proportion of HPV16-positive women with low-grade lesions progress to CIN2+, a methylation test with PPV of 44 and 90% NPV could help triage women and identify those at lower risk who could be followed-up less frequently. Validation is a sequential process and additional validations are required before one may consider our assay to be qualified for clinical use. In addition, a cutoff needs to be validated in comparable populations with standardized sample collection and storage. If confirmed by other studies and extended to different populations, the methylation classifier could lead to an advance in the triage of women positive for HPV16 DNA and could potentially be extended to other HPV types. The methylation patterns of several other HPV types have recently been reported to be similar to HPV16 encouraging the possibility of developing a HR-HPV group methylation test.^24^ Our study provides a model for the study of methylation of other HPV types and suggests the HPV methylation test as a possible second molecular test on top of the available HR-HPV tests to triage HPV infected women for colposcopy. All unmethylated HPV16 infections could be treated as of lower risk but still be more frequently called for repeat testing than HPV16 negative women. In comparison to cytology, the sensitivity of the S1 classifier was higher (92 *vs*. 67%) while the specificity was similar (42 *vs*. 40%). This suggests that the methylation-based classifier score may be preferred for triage if confirmed in larger studies.

There are several ongoing studies in different populations of women from Costa Rica, the USA and Europe that are evaluating the potential value of epigenetic testing for cervical cancer. This new approach to triage may become more important when HPV DNA testing becomes the primary screening method. Most assays to test for DNA methylation are still in the research realm but some such as quantitative methylation-specific PCR (qMSP) have made the transition to clinical utility^25^,^26^; however, we did not choose the qMSP assay because pyrosequencing (PSQ) may be a simpler and more accurate test for quantitative HPV CpG methylation measurements.^13^,^20^,^27^ PSQ is inherently quantitative as it takes the ratio of *C* to *C* + *T* in CpG sites that are treated with sodium bisulfite reagent. Bisulfite converts all nonmethylated *C*'s to *T*'s and these changes can be precisely measured by the pyrosequencing method and compared to internal controls. There is no need to take ratios to external controls such as for qMSP that may lead to potential problems of increased variability. Although pyrosequencing-based measurement of DNA methylation is still somewhat in the research realm, it appears ready for the transition to a more clinical setting, and furthermore, simpler methylation assays could be developed, perhaps based on high-resolution melt analyses or next generation sequencing.^28^,^29^ It appears that quantitative DNA methylation testing of HPV in routine settings may be feasible given sufficient attention from developers of diagnostic tests.

There are numerous human genes, MAL, CADM1, hsa-mir-124 *etc*. that appear to have potential for triage of women with HR-HPV infections or abnormal cytology.^25^,^26^,^30^ One of the more promising to date are a combination of MAL and CADM1 which could be a possible triage test for HR-HPV screening, giving a reported sensitivity of 87% and specificity of 43%.^26^ In the same set of women, the triage sensitivity and specificity values for cytology were 66 and 79%, respectively, while cytology combined with genotyping for HPV16 and HPV18 had a sensitivity of 84% and a specificity of 54%.^26^ Use of human genes as DNA methylation diagnostic targets has the advantage of being non-HPV type specific and if combined with an HPV DNA methylation test, may be a source of an effective triage tool to detect women at high risk of developing CIN2+. A study of a combination test is underway.

To reach 90% sensitivity with highest possible specificity, the cutoff value in S1 was adjusted to 67 in the UK cohort, compared to 5 in the Costa Rica cohort. The large difference in the cutoff, may be due to a number of factors, such as differences between the populations, study size or storage of the specimens in different media—SurePath in the UK and STM (standard transport medium) in the Costa Rican cohort, respectively. It is also possible that the methylation risk classifier may have different forms and cutoffs depending on the specific clinical application.

A potential limitation to our study is that the validation of the classifier score was performed in women with low-grade cytology, which is not the target population for intended use in a setting for triage of primary HPV16 DNA-positive women. Although not a screening population, the advantage of our cohort was the high disease rate, allowing a more accurate evaluation of sensitivity and specificity in this relatively small study. Furthermore, the classifier score was developed on methylation data obtained from Costa Rican women who entered the study without any evidence of cervical disease and then followed for years without any evident cervical abnormalities until the incident CIN2+ were detected. The Costa Rican population used to derive the risk scores is ethnically different to the UK cohort and the fact that the scores worked similarly in both populations of women from different continents is encouraging. Another limitation is the relatively small sample size of our study and thus the rather wide confidence intervals. Larger studies on different populations of women are necessary to show that methylation testing of the HPV genome is a robust and feasible test in the routine setting. Expansion of methylation panels to other HR-HPV types is also desirable.

In conclusion, a predefined classifier score based on HPV16 methylation in L1 and L2 in a Central American population was found to have similar differentiating potential to identify CIN2/3 in European women. Our successful validation of the predefined classifiers indicates the generality of our approach to test for DNA methylation in the L1 and/or the L2 regions of HPV16.

## Abbreviations

AUC: area under the ROC curve
C: cytosine
CIN: cervical intraepithelial neoplasia
CIN2+: CIN grade 2 or worse
DIM: diindolylmethane
E6: early region 6
HPV16: human papillomavirus type 16
HR-HPV: high-risk human papillomavirus
IQR: interquartile range
L1: Late region 1
L2: late region 2
NPV: negative predictive value
ORF: open reading frame
PCR-EIA: PCR-enzyme immunoassay
PPV: positive predictive value
PSQ: pyrosequencing
ROC: Receiver operating characteristic
T: thymidine
URR: upstream regulatory region
