# Supplementary material for: HPV16 L1 and L2 DNA methylation predicts high-grade cervical intraepithelial neoplasia in women with mildly abnormal cervical cytology
Source: Int J Cancer. 2013 Jan 21;133(3):637–44. doi: 10.1002/ijc.28050 (PMC3708123; doi:10.1002/ijc.28050)

**Supplementary Table 1.** Selected ROC sensitivity (Se), specificity (Sp), positive predictive value (PPV) and negative predictive value (NPV) from the S1 classifier to predict CIN2/3 at the six-month follow-up. T+/- is HPV16 test positive or negative; C+/- is CIN2/3 negative or positive counts.

| **S1** |  | **T+C+** | **T-C+** | **T+C-** | **T-C-** |  | **Se.** | **Sp.** | **PPV** | **NPV** |
| --- | --- | --- | --- | --- | --- | --- | --- | --- | --- | --- |
| 0.0 |  | 25 | 0 | 48 | 0 |  | 100% | 0% | 34% |  |
| 2.4 |  | 24 | 1 | 47 | 1 |  | 96% | 2% | 34% | 50% |
| 3.2 |  | 24 | 1 | 45 | 3 |  | 96% | 6% | 35% | 75% |
| 3.8 |  | 24 | 1 | 43 | 5 |  | 96% | 10% | 36% | 83% |
| 5.0 |  | 24 | 1 | 41 | 7 |  | 96% | 15% | 37% | 88% |
| 9.8 |  | 24 | 1 | 39 | 9 |  | 96% | 19% | 38% | 90% |
| 41.0 |  | 24 | 1 | 35 | 13 |  | 96% | 27% | 41% | 93% |
| 66.2 |  | 23 | 2 | 31 | 17 |  | 92% | 35% | 43% | 89% |
| 66.6 |  | 23 | 2 | 29 | 19 |  | 92% | 40% | 44% | 90% |
| 67.4 |  | 21 | 4 | 28 | 20 |  | 84% | 42% | 43% | 83% |
| 68.0 |  | 20 | 5 | 24 | 24 |  | 80% | 50% | 45% | 83% |
| 68.8 |  | 19 | 6 | 20 | 28 |  | 76% | 58% | 49% | 82% |
| 69.4 |  | 19 | 6 | 15 | 33 |  | 76% | 69% | 56% | 85% |
| 70.4 |  | 17 | 8 | 12 | 36 |  | 68% | 75% | 59% | 82% |
| 71.6 |  | 14 | 11 | 10 | 38 |  | 56% | 79% | 58% | 78% |
| 73.0 |  | 12 | 13 | 7 | 41 |  | 48% | 85% | 63% | 76% |
| 73.2 |  | 12 | 13 | 6 | 42 |  | 48% | 88% | 67% | 76% |
| 74.0 |  | 11 | 14 | 6 | 42 |  | 44% | 88% | 65% | 75% |
| 74.4 |  | 10 | 15 | 6 | 42 |  | 40% | 88% | 62% | 74% |
| 74.6 |  | 9 | 16 | 6 | 42 |  | 36% | 88% | 60% | 72% |
| 74.6 |  | 8 | 17 | 6 | 42 |  | 32% | 88% | 57% | 71% |
| 75.0 |  | 8 | 17 | 5 | 43 |  | 32% | 90% | 62% | 72% |
| 75.2 |  | 8 | 17 | 4 | 44 |  | 32% | 92% | 67% | 72% |
| 76.0 |  | 7 | 18 | 4 | 44 |  | 28% | 92% | 64% | 71% |

Supplementary Table 2. Selected ROC points for the S3 classifier to predict persistence of HPV16 infection using baseline methylation measurements in 82 women. T+ and T- are HPV16 test positive or negative respectively; C+ and C- are presence or absence of CIN2/3 respectively; PPV is positive predictive value and NPV is negative predictive value.

| **S3** | **T+C+** | **T-C+** | **T+C-** | **T-C-** | **Sensitivity** | **Specificity** | **PPV** | **NPV** |
| --- | --- | --- | --- | --- | --- | --- | --- | --- |
| 0 | 71 | 0 | 11 | 0 | 100% | 0% | 87% |  |
| 1.7 | 68 | 3 | 10 | 1 | 96% | 9% | 87% | 25% |
| 2.7 | 68 | 3 | 9 | 2 | 96% | 18% | 88% | 40% |
| 3.9 | 68 | 3 | 8 | 3 | 96% | 27% | 89% | 50% |
| 4.6 | 67 | 4 | 8 | 3 | 94% | 27% | 89% | 43% |
| 6 | 66 | 5 | 8 | 3 | 93% | 27% | 89% | 38% |
| 7.3 | 63 | 8 | 8 | 3 | 89% | 27% | 89% | 27% |
| 7.5 | 63 | 8 | 6 | 5 | 89% | 45% | 91% | 37% |
| 8 | 60 | 11 | 6 | 5 | 85% | 45% | 91% | 31% |
| 9.2 | 57 | 14 | 6 | 5 | 80% | 45% | 90% | 26% |
| 10.7 | 51 | 20 | 5 | 6 | 72% | 55% | 91% | 23% |
| 11.1 | 50 | 21 | 4 | 7 | 70% | 64% | 93% | 25% |
| 12 | 47 | 24 | 4 | 7 | 66% | 64% | 92% | 23% |
| 13.2 | 42 | 29 | 4 | 7 | 59% | 64% | 91% | 19% |
| 14.5 | 39 | 32 | 2 | 9 | 55% | 82% | 95% | 22% |
| 16.3 | 34 | 37 | 2 | 9 | 48% | 82% | 94% | 20% |
| 18.6 | 29 | 42 | 2 | 9 | 41% | 82% | 94% | 18% |
| 22.1 | 24 | 47 | 2 | 9 | 34% | 82% | 92% | 16% |
| 25.5 | 19 | 52 | 2 | 9 | 27% | 82% | 90% | 15% |
| 28.5 | 15 | 56 | 1 | 10 | 21% | 91% | 94% | 15% |
| 38 | 10 | 61 | 1 | 10 | 14% | 91% | 91% | 14% |
| 44.6 | 5 | 66 | 1 | 10 | 7% | 91% | 83% | 13% |
| 48.5 | 4 | 67 | 1 | 10 | 6% | 91% | 80% | 13% |
| 50.5 | 4 | 67 | 0 | 11 | 6% | 100% | 100% | 14% |
| 53.7 | 3 | 68 | 0 | 11 | 4% | 100% | 100% | 14% |
| 61.8 | 2 | 69 | 0 | 11 | 3% | 100% | 100% | 14% |
| 68.9 | 1 | 70 | 0 | 11 | 1% | 100% | 100% | 14% |

Supplementary table 3. Cross-tabulation of cytology result and CIN status in 73 women who were HPV16 positive at six months.

|  |  | Histology | |
| --- | --- | --- | --- |
| Cytology | Total N | <CIN2  N (%) | CIN2/3  N (%) |
| Normal | 26 | 18 (69) | 8 (31) |
| Borderline | 13 | 11 (85) | 2 (15) |
| Mild | 14 | 10 (61) | 4 (29) |
| Moderate | 9 | 3 (33) | 6 (67) |
| Severe | 4 | 0 (0) | 4 (100) |
| Inv SCC^1^ | 1 | 1 (100) | 0 (0) |
| Total | 67 | 43^2^ | 24 |

^1^ Invasive squamous cell carcinoma

^2^ There were 5 missing values in the <CIN2 group and 1 missing value in the CIN2/3 group

**Supplementary Figure 1.** Change in methylation by CpG site, from all 84 pairs of samples at baseline and six months, where failed methylation assays are imputed as zero methylation.


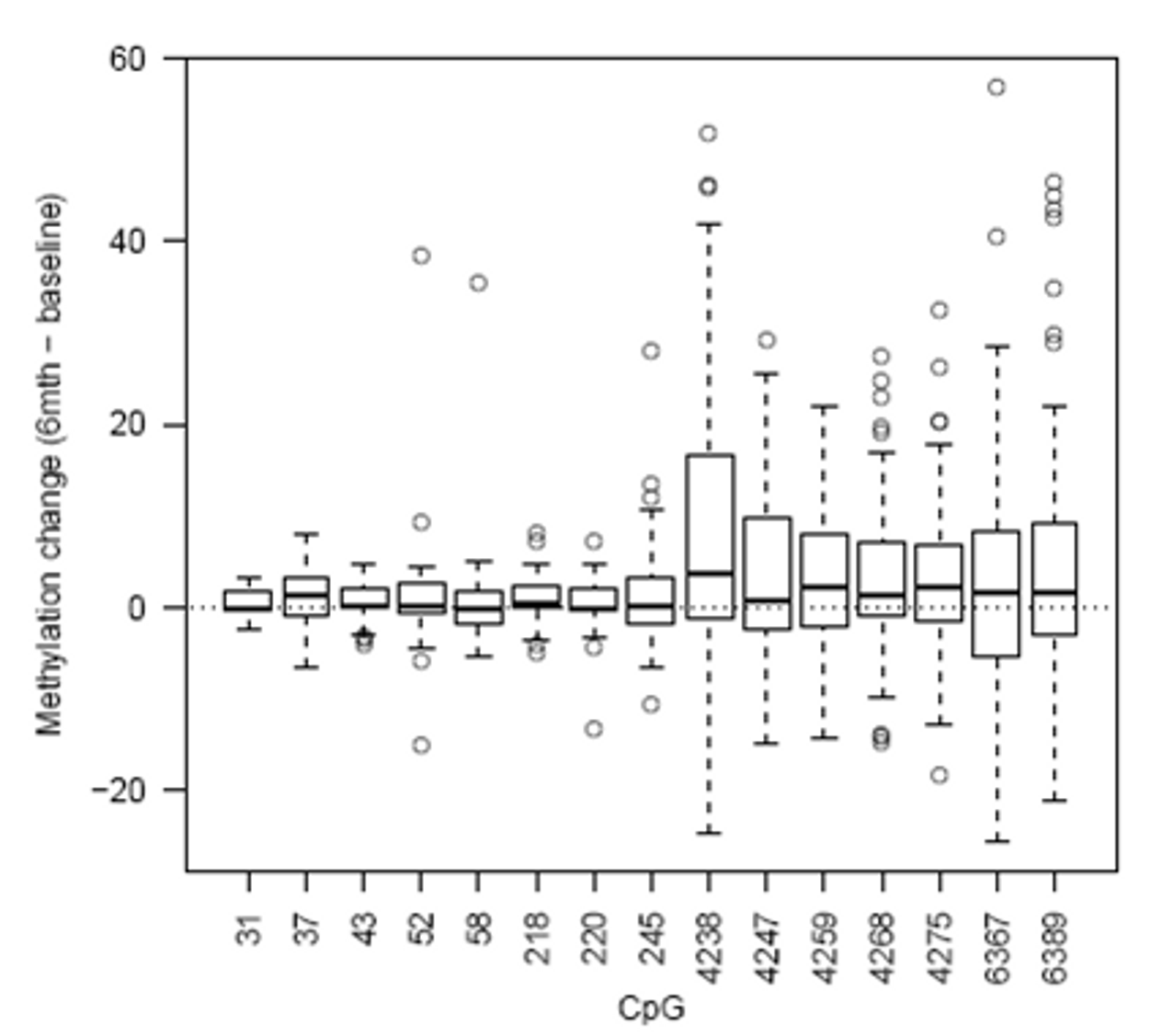

Supplement: Supplementary file 1 [file ijc0133-0637-SD1.doc]
