# Supplementary material for: HPV16 L1 and L2 DNA methylation predicts high-grade cervical intraepithelial neoplasia in women with mildly abnormal cervical cytology
Source: Int J Cancer. 2013 Jan 21;133(3):637–44. doi: 10.1002/ijc.28050 (PMC3708123; doi:10.1002/ijc.28050)
Supplement: Supplementary file 2 [file ijc0133-0637-SD2.pdf]

Fitted HPV-16 DNA methylation classifiers in a Costa Rican cohort

Attila T Lorincz, Adam R Brentnall, Natasa Vasiljevic, Dorota Scibior-Bentkowska, Alejandra Castanon, Alison Fiander, Ned Powell, Amanda Tristram, Jack Cuzick, Peter Sasieni

October 24, 2012

This brief report outlines classification scores developed to identify high risk women who have human papillomavirus type 16 (HPV-16) using DNA methylation data from a study in Costa Rica (Mirabello et al., 2012). Fitted scores and their sensitivity and specificities are given. They were obtained before analysis of methylation data from CRISP.

1 Data and methods

The data from Costa Rica were DNA methylation measurements on 172 individuals, composed of 92 who cleared, 36 who persisted and 44 who progressed to CIN2/3. Selected measurements were made in five categories, namely methylation

1. just before clearance;
2. for HPV-16 persistence over a shorter period (persister first sample);
3. for HPV-16 persistence over a longer period (persister last sample);
4. for CIN2/3 diagnosed in the future (CIN2/3 first sample); and
5. for CIN2/3 diagnosed around the same time as the sample (CIN2/3 last sample).

Missing methylation measurements were imputed to be zero for derived variables that count the number of zero CpGs, because data analysis shows that missing CpG sites in a gene or region were more likely to jointly occur with zero methylation in other CpGs. For L1 the mean methylation was used, but missing values were not imputed to be zero unless all CpGs were missing.

Summary tables are first used to show the overall structure of the data in an exploratory analysis. Then the scores are fitted using logistic regression models to predict development to CIN2/3, or persistence. Receiver operating characteristic (ROC) plots and tables show fitted sensitivity and specificities.

2 Results

2.1 Exploratory analysis

The main patterns from the CRISP CpGs in Costa Rica are shown in Tables 1 and 2. In summary,

Table 1: Summary statistics for the markers, where L1 is median methylation (%); L2, URR and E6 are percent methylated.

		L1	L2	URR	E6
Clear		9	29	40	51
Persist	Shorter	23	24	39	43
Persist	Longer	25	68	42	53
CIN2/3	Eventually	27	46	61	57
CIN2/3	Now	30	77	75	86

Table 2: Graphical representation of Table 1, where + denotes elevated; ± in L2 is midway between - and +.

		L1	L2	URR	E6
Clear		-	-	-	-
Persist	Shorter	+	-	-	-
Persist	Longer	+	+	-	-
CIN2/3	Eventually	+	±	+	-
CIN2/3	Now	+	+	+	+

Table 3: Classifier 1, primary model. Selected ROC points with score cutoff.

Score	sens	spec
0.00	1.00	0.00
0.09	0.95	0.37
0.12	0.95	0.41
0.18	0.95	0.45
0.22	0.95	0.49
0.25	0.93	0.52
0.29	0.86	0.54
0.37	0.86	0.58
0.45	0.86	0.62
0.59	0.86	0.66
0.67	0.84	0.69
0.79	0.84	0.73
0.87	0.82	0.76
1.04	0.80	0.80
1.34	0.77	0.83
1.51	0.70	0.84
1.74	0.66	0.87
2.16	0.61	0.89
2.34	0.55	0.91
2.86	0.55	0.95
3.27	0.45	0.95
3.70	0.36	0.96
3.89	0.27	0.98
4.01	0.20	0.99
4.29	0.11	1.00

- L1 appeared useful to show whether a case is just about to clear.
- L2 might be linked to length of persistence.
- URR might be linked to CIN2/3, now and in the future.
- E6 might be linked to CIN2/3 now.

2.2 Classifier 1: diagnose disease

Primary classifier

The comparison between samples (1. clearance and 2. persister first sample) *vs* (5. CIN2/3 last sample) is used to fit the first classification rule. The strongest variables identified in an exploratory analysis were

- proportion of CRISP L2 CpG sites methylated (x_1);
- mean methylation of L1 (x_2).

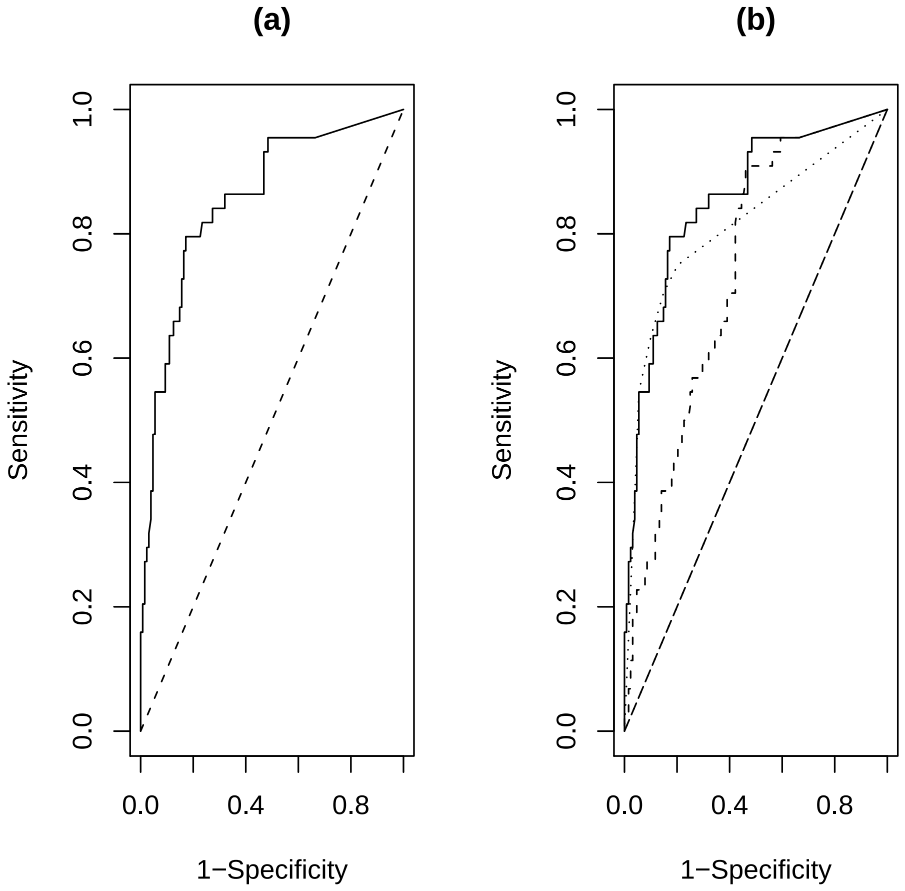

Figure 1: Classifier 1, primary model. ROC plots for the (a) fitted model, and (b) the fitted model (—), L2 only (. . .), L1 only (- - -).

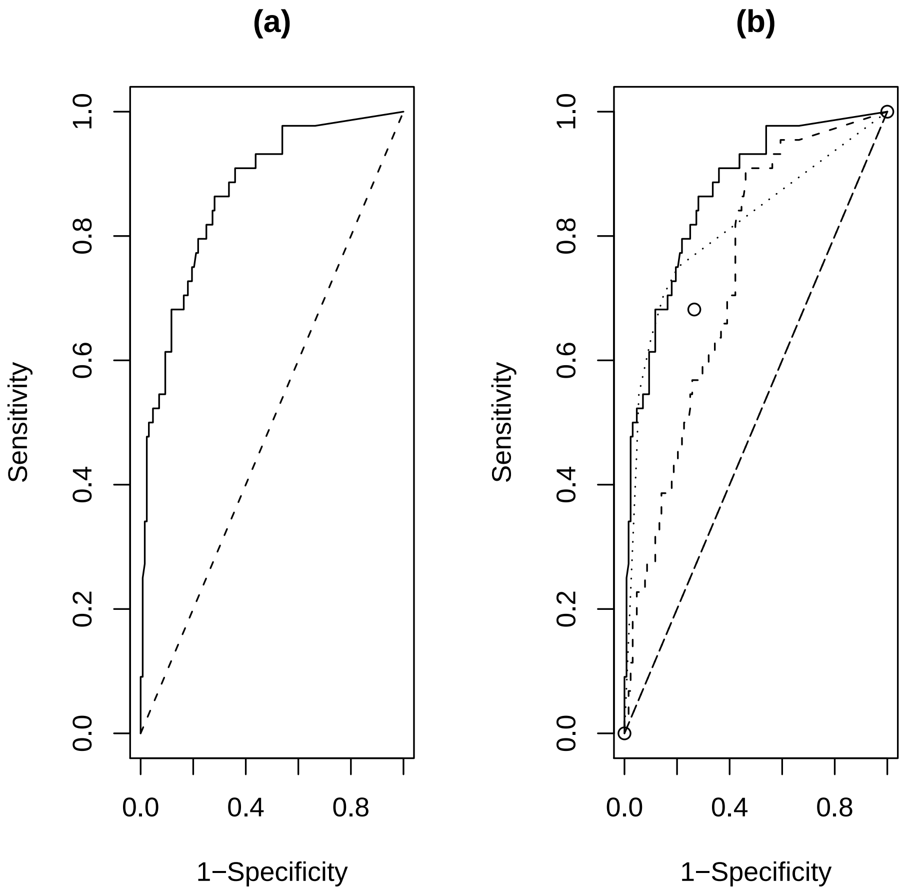

Figure 2: Classifier 1, secondary model. ROC plots for the (a) fitted model, and (b) the fitted model (—), L2 only (. . .), L1 only (- - -) and URR/E6 methylated (o).

The fitted score from a logistic regression model was

$$3.17x_1 + 1.83x_2$$

Thus it runs from zero to a maximum 5, but can be re-scaled to run between 0 and 100, say. Figure 1a shows the ROC from the fitted model. The components are plotted in Figure 1b. This shows that L2 contributes the most, due to the relatively large difference between the two groups in Table 1. However, L1 might add information that is useful for high sensitivity. Table 3 shows some ROC points.

Secondary classifier

The secondary model adds URR and E6 to L1 and L2. Table 5 suggests that E6 and URR might be used together: when both were methylated it was more likely that a women progressed to disease. The following variables were therefore used in a logistic regression.

- Proportion of CRISP L2 CpG sites methylated (x_1);
- Mean methylation of L1 (x_2); and
- Indicator variable for both E6 and URR with at least one CpG methylated (x_3).

The fitted score was

$$2.65x_1 + 2.23x_2 + 1.1x_3.$$

Table 4: Classifier 1, secondary model. Selected ROC points with score cutoff.

Score	sens	spec
0.00	1.00	0.00
0.12	0.98	0.38
0.20	0.98	0.41
0.30	0.98	0.45
0.41	0.93	0.48
0.56	0.93	0.52
0.74	0.93	0.55
0.93	0.91	0.59
1.09	0.91	0.62
1.22	0.89	0.66
1.33	0.86	0.69
1.40	0.84	0.72
1.58	0.82	0.75
1.76	0.80	0.78
1.97	0.73	0.80
2.12	0.70	0.84
2.40	0.68	0.87
2.65	0.61	0.88
3.00	0.57	0.91
3.32	0.52	0.93
3.52	0.50	0.96
3.95	0.43	0.98
4.33	0.34	0.98
4.62	0.23	0.99
4.80	0.11	0.99

Table 5: Number of CIN2/3 cases by URR and E6 status, which are said to be methylated if any one CpG site has non-zero methylation.

	Neither	URR only	E6 only	Both
Number	3 / 57	3 / 19	8 / 32	30 / 64
Percent	5	16	25	47

Table 6: Contingency table for E6/URR and L2 methylation, Clear/Persist

	Not E6 and URR	E6 and URR	(Perc E6 & URR)
L2 unmethylated	84	18	18
L2 methylated	10	16	62

Table 7: Contingency table for E6/URR and L2 methylation, CIN2/3

	Not E6 and URR	E6 and URR	(Perc E6 & URR)
L2 unmethylated	6	5	45
L2 methylated	8	25	76

Thus the score runs from zero to a maximum 6. Figure 2a shows the ROC from the fitted model. The components are plotted in Figure 2b. Table 4 shows selected ROC points. Contingency Tables 6 and 7 identify where individuals fall in cross tabulations of L2 and URR/E6 variables depending on their outcome. Although limited, these data indicate that E6/URR might be useful after allowing for L2. Finally, it was observed that L1 methylation was higher on average when URR and E6 were methylated (26% against 19%).

2.3 Classifier 2: predict persistance from clearance

We compare samples (1. clearance) and (2. persister first). For this Table 1 suggests that L1 is the only gene/region of use. Table 8 shows that classifying all with mean methylation of more than 4% as persisters had approximately 69% sensitivity for approximately 38% specificity.

References

Mirabello, L., C. Sun, A. Ghosh, A. C. Rodriguez, M. Schiffman, N. Wentzensen, A. Hildesheim, R. Herrero, S. Wacholder, A. Lorincz, and R. D. Burk (2012). Methylation of human papillomavirus type 16 genome and risk of cervical precancer in a costa rican population. *Journal of the National Cancer Institute 104*(7), 556–565.

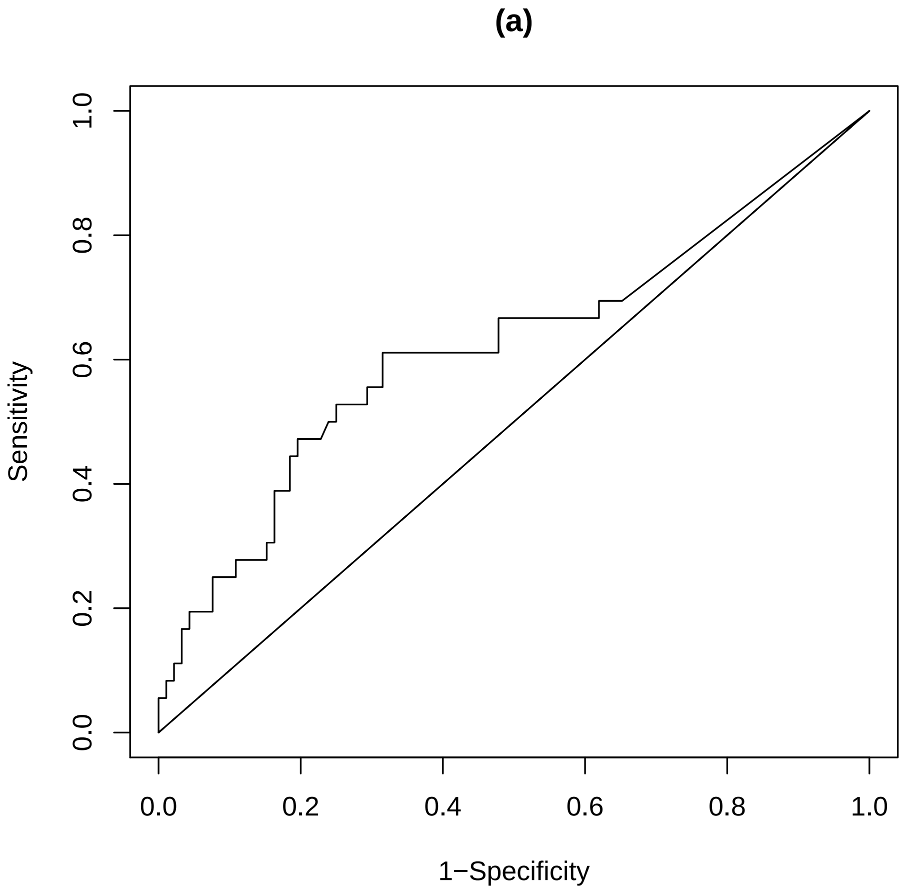

Figure 3: Classifier 2. ROC to predict clearance from persistence (first) using L1 alone.

Table 8: Classifier 2. Selected ROC points with L1 cutoff.

L1	sens	spec
0.00	1.00	0.00
0.04	0.69	0.35
0.04	0.69	0.36
0.04	0.69	0.37
0.04	0.69	0.38
0.05	0.67	0.38
0.05	0.67	0.39
0.05	0.67	0.41
0.05	0.67	0.42
0.06	0.67	0.43
0.06	0.67	0.46
0.10	0.67	0.51
0.11	0.61	0.55
0.13	0.61	0.61
0.16	0.61	0.67
0.20	0.56	0.71
0.22	0.53	0.75
0.25	0.47	0.79
0.30	0.39	0.82
0.32	0.31	0.84
0.36	0.28	0.88
0.42	0.25	0.92
0.45	0.19	0.96
0.51	0.11	0.98
